# Review on the Selection of Aptamers and Application in Paper-Based Sensors

**DOI:** 10.3390/bios13010039

**Published:** 2022-12-27

**Authors:** Kaifei Wang, Minglu Wang, Teng Ma, Wenyu Li, Hongyan Zhang

**Affiliations:** 1 Key Laboratory of Food Nutrition and Safety of Shandong Normal University, College of Life Sciences, Shandong Normal University, Jinan 250358, China; 2 Shandong Provincial Key Laboratory of Animal Resistance Biology, Shandong Normal University, Jinan 250014, China

**Keywords:** aptamer, paper-based sensor, SELEX, test strip and detection method

## Abstract

An aptamer is a synthetic oligonucleotide, referring to a single-stranded deoxyribonucleic acid or ribonucleic acid ligand produced by synthesis from outside the body using systematic evolution of ligands by exponential enrichment (SELEX) technology. Owing to their special screening process and adjustable tertiary structures, aptamers can bind to multiple targets (small molecules, proteins, and even whole cells) with high specificity and affinity. Moreover, due to their simple preparation and stable modification, they have been widely used to construct biosensors for target detection. The paper-based sensor is a product with a low price, short detection time, simple operation, and other superior characteristics, and is widely used as a rapid detection method. This review mainly focuses on the screening methods of aptamers, paper-based devices, and applicable sensing strategies. Furthermore, the design of the aptamer-based lateral flow assay (LFA), which underlies the most promising devices for commercialization, is emphasized. In addition, the development prospects and potential applications of paper-based biosensors using aptamers as recognition molecules are also discussed.

## 1. Aptamers

Aptamers are single-stranded deoxyribonucleic acid (ssDNA) or ribonucleic acid ligands produced by exponential enrichment, and capable of binding to small molecules and macromolecules, such as proteins or cells, with high affinity and specificity [1,2,3,4,5,6,7,8,9]. Tuerk and Gold designed an RNA library containing variant sequences with eight random nucleotides at specific sites in which they obtained two RNA sequences with high affinity and specificity by using T4 DNA polymerase as a target [10]. This random library contained 48 different sequences, each of which had an equal chance to contact the target. The number of high-affinity sequences was increased through multiple rounds of screening of candidate sequences until they dominated the library. This process was named the Systematic Evolution of Ligands by Exponential Enrichment (SELEX). In the same year, Ellington and Szostak carried out similar work, and a library of 1010 different sequences for RNA sequences that specifically bound to small organic dyes was screened. They named the high-affinity sequences “aptamers.” The word “aptamer” is a combination of the Latin word aptus (to fit) and the Greek word meros (part) [11].

As a new type of recognition elements for biosensors, aptamers have some advantages over antibodies. They are non-animal derived, easier to synthesize, smaller, purer, more stable amid temperature changes, and more biologically active [12,13]. Aptamers can recognize a wide range of molecules, and serve as promising candidates for antibodies, having the advantages of short screening cycles, low cost, and long-term preservation [14]. The length of an aptamer usually varies from 10 to 100 bases [13], with typical structural motifs such as stems, internal loops, purine-rich bulges, hairpin structures, pseudo knots, kissing complexes, and G-quadruplex structures. Furthermore, aptamers exhibit greater flexibility for designing different detection modes because they are composed of nucleic acids that hybridize between molecules and have direct sequence-determining properties [15].

To date, aptamers have been screened for different targets, including small organic compounds [16], proteins [17], and cells [18], with high affinity after undergoing adaptive conformational changes and specific three-dimensional folding [15].

### 1.1. Aptamer Screening Technologies

Aptamers are primarily involved in nucleic acid aptamers. These are high-affinity short RNA or single-stranded DNA (ssDNA) oligonucleotides screened by SELEX technology [10,11]. SELEX is a cyclical process that exploits the specific affinities between ssDNAs with various intramolecular structures and the target, and a typical SELEX process involves incubation, isolation, and amplification. First, an original oligonucleotide library consisting of random sequences and constant primers is created [10]. The original library is then incubated with the target of interest to generate a large number of ssDNA lines. The aptamers have abundant structures and installations to ensure that the aptamer binding to the target is accomplished [19]. The screened sequences are subsequently amplified by polymerase chain reaction (PCR). Then, the screening and amplification processes are repeated until the binding sequences dominate the library. Finally, the screened oligonucleotides are sequenced and analyzed.

Traditional SELEX techniques are tedious and time-consuming. In the modern era, considerable research efforts have been undertaken to improve SELEX technology and optimize the process. Many scientists working on SELEX technology have discovered various techniques that can retain the advantages of SELEX technology while simplifying the screening process for efficiency. They are summarized in Table 1.

#### 1.1.1. Magnetic Bead-SELEX (Mag-SELEX)

In 2005, Stoltenburg et al. created the fluorescently labeled magnetic bead-SELEX (Mag-SELEX) based on the classical SELEX screening method [20]. Mag-SELEX uses magnetic beads to immobilize ssDNAs or targets and isolates the aptamers from the targets in a magnetic field. Small molecules are immobilized on the surface of the magnetic beads, and the unbound oligonucleotides and target-bound aptamers are separated, thus significantly reducing time.

Mag-SELEX technology is the most common technique in aptamer screening due to its simplicity of operation, high separation efficiency, and wide target range. However, the screening cycle of this technique is long, usually requiring 5–14 rounds with high reagent consumption. Moreover, after the target is immobilized, a steric hindrance to the target is generated, thus affecting the binding of ssDNAs to the target and resulting in the lowered affinity and specificity of the screened aptamers.

#### 1.1.2. Capture-SELEX

Capture-SELEX can be applied for aptamer screening of solute targets, instead of immobilizing a target, such as small molecules, on a solid surface. In Capture-SELEX, a random library consisting of long and continuous sequences is constructed first and then immobilized on a carrier. The immobilized sequence undergoes a conformational change upon binding to a free target in solution, thus releasing itself from the sequence-carrier complex. It is subsequently separated by simple approaches, such as magnetic separation or centrifugation, and high affinity and specificity aptamers are thereby obtained [21]. This method maximizes the retention of the spatial conformation and binding sites of small molecule targets.

**Table 1:** Summary of aptamer screening technologies.

Methods	Description	Advantages	Disadvantages	Ref.
Mag-SELEX	Targets or ssDNAs are coupled to the surfaces of magnetic beads by a chemical coupling reaction, and the binding sequences are separated by a magnetic field.	Convenient and rapid magnetic separation. Various magnetic bead surface modifications for efficient target fixation, such as modifications using carboxyl, amino, and streptavidin.	Few functional groups available for coupling in some molecules. Complicated coupling process.	[21]
Capture-SELEX	A short-chain nucleotide is designed as a bridging sequence and is located on a solid phase carrier. The oligonucleotide library is fixed on the carrier based on the principle of base complementary pairing. When the target specifically recognizes the nucleotide sequence, the conformation of the oligonucleotide library is induced to change, and then the oligonucleotide library is detached from the solid phase carrier.	Long random sequences in oligonucleotide libraries that can form complex structures and bind targets with high specificity. A superior design that facilitates the conformational changes of aptamers and induces the oligonucleotide sequences to detach from the solid carrier.	Low screening efficiency due to the “false positive” result that is caused by the dissociation from targets of unbound sequences in immobilized libraries. Low abundance and diversity of libraries during the screening process. Complex and non-uniform equipment.	[22]
CE-SELEX	The bound and unbound oligonucleotide sequences are separated in a free solution according to their mobility differences.	Highly efficient separation. High selectivity. Low screening round numbers (generally only 2–4 rounds), and this leads to a high enrichment rate of the library and greatly improves the screening efficiency.	Not suitable for target molecules without high electrophoretic mobilities and commonly used for aptamer screening of large targets. High screening cost due to the expensive capillary electrophoresis apparatus.	[23]
GO-SELEX	Free ssDNAs or RNAs are adsorbed by the π–π stacking interaction, while oligonucleotides with structural changes, such as aptamer-target complexes, are not absorbed.	Effective guarantee of the natural conformations of targets or oligonucleotides. Reduction in the spatial impedance (no fixed-induced structural changes). Less non-specific adsorption of the solid substrate used to fix targets or libraries.	Potential self-desorption of the oligonucleotide sequences adsorbed on the GO surface by interaction. This results in an increase in non-specific sequences. Not suitable for aptamer screening of target molecules that can be adsorbed by GO.	[24]
Microfluidic chip SELEX	A SELEX technology based on a microfluidic system: a micro platform capable of automated phylogenetic screening of ligands on a single-chip basis.	Small device. Small sample size. Automatic screening.	Unstable injection volume. Limitation of the hydrophilicity of the microchannel. Not commercially available and time-consuming to build.	[25]

#### 1.1.3. Capillary Electrophoresis-SELEX (CE-SELEX)

Capillary electrophoresis-SELEX (CE-SELEX) is a process in which aptamer screening occurs in capillary tubes. Compared with conventional SELEX, CE-SELEX requires less time, has no spatial obstruction, and can be performed on non-fixed targets. Moreover, CE-SELEX uses minimal amounts of samples and solvents, which can reduce the use of chemicals.

CE-SELEX is performed by first incubating the target with a random library, followed by electrophoresis. Depending on whether the sequence is bound to the target or not, its charge-to-mass ratio varies, and the electric field migration rate changes accordingly. Based on the electrophoretic mobility, the aptamers are isolated and screened. The advantage of this method is that the bound sequences can be quickly isolated from the unbound ones, and it usually only takes 2–4 rounds to screen aptamers with high specificity and affinity [26]. Therefore, the screening cycle is shortened. However, this technique is expensive and requires complex instrumentation. For aptamer screening, the cost is high. Therefore, the use of CE-SELEX to screen aptamers of high molecular mass targets is limited [27].

#### 1.1.4. Graphene Oxide-SELEX (GO-SELEX)

Graphene oxide-SELEX (GO-SELEX) is a non-immobilized SELEX that does not require immobilization of the target and the library. The GO is an emerging two-dimensional carbon nanomaterial that can adsorb ssDNAs to its surface through π-π stacking. GO-SELEX does not require the use of a dedicated instrument and only requires centrifugation to achieve separation of the aptamers from GO. The target is incubated with the ssDNA library, and then GO is added. GO adsorbs free ssDNAs which are not bound to the target. Because the ssDNA-target complexes are not centrifugated into the sediment, the separation between ssDNA-target complexes and non-adsorbed free ssDNAs can be achieved by centrifugation [28].

#### 1.1.5. Microfluidic Chip SELEX

A microfluidic system is a micro platform capable of automated phylogenetic screening of ligands on a single chip basis. Compared with traditional SELEX methods, microfluidic SELEX technology uses smaller equipment and requires less sample volume. Aptamers can be screened automatically on an integrated device that includes incubation, extraction, and amplification operations, thus automating the SELEX process.

The microfluidic system includes a microfluidic module, micropumps, microvalves, reagent loading chambers, temperature control modules, a transfer unit, a waste chamber, and a PCR chamber. This configuration ensures that multiple rounds of extraction and amplification can be completed quickly.

### 1.2. Optimization Strategy of the Nucleic Acid Aptamer Sequence

The nucleic acid aptamers obtained by SELEX technology often suffer from long sequences and low affinity, so further optimization is needed for practical applications. Current methods for modification of nucleic acid aptamers include chemical modification, truncation, cleavage, bivalent or multivalent construction, and random or site-specific mutagenesis [29]. This section summarizes the truncation, cleavage, and splicing of nucleic acid aptamer sequences.

#### 1.2.1. Truncation of Nucleic Acid Aptamer Sequences

The screened aptamers (full-length aptamers) are typically 80–100 nucleotides. However, not all nucleotides in the full-length aptamer play a critical role in binding to the target. A full-length aptamer typically has three functional regions. The region that is responsible for binding to the target is approximately 10–15 nt [30]. This region has different secondary structures and usually contains hairpin loops, G-quadrilateral loops, bumps, or pseudoknots. Another region contains nucleotides that do not directly bind to the target but play an important role in target-aptamer binding [30]. Generally, the number of basic nucleotides in this region is about 25–40 nt [31]. The third region includes nucleotides that neither bind to the target nor support the target-aptamer binding. These nucleotides are considered nonessential nucleotides. Following the aptamer selection process, it is always desirable to truncate the aptamer to eliminate nonessential nucleotides [32]. Minimizing the nucleic acid aptamer length can effectively reduce the cost of synthesis, allow for more flexibility in sensor design, and provide higher affinity and selectivity.

#### 1.2.2. Cleavage of Nucleic Acid Aptamer Sequences

In target-aptamer binding, targets with small molecular weights generally contain only one specific binding site or a part of a binding site, so steric hindrance may prevent two recognition units from simultaneously binding to the target [33]. To solve this problem, Stojanovic et al. proposed a strategy to cleave the nucleic acid aptamer into two fragments that can form a specific ternary complex only in the presence of the target [16]. Compared with the intact aptamer, the cleaved aptamer provides a lower non-specific signal [34].

Currently, cleaved aptamers are generally found in three types: hairpin-loop structures, three-way linkage structures, or those that bind to targets through specific binding mechanisms. Among them, the bases not involved in target-aptamer bindings are generally used as cleavage sites for aptamers in known systems. In addition, aptamers with hairpin loops or three-way linkage structures are susceptible to being cleaved into two or more fragments. They usually contain two or three binding regions, and small molecule targets typically bind to the hairpin-loop regions [35] or the central part of three-way linkage structures. When these aptamers are cleaved, the loops or stems near the main area are generally retained, and the number of binding bases in the resulting branches is then sequentially optimized.

#### 1.2.3. Splicing of Nucleic Acid Aptamer Sequences

Aptamers may have the disadvantage of weak binding with their targets [36], so constructing multivalent nucleic acid aptamers is an effective strategy for improving the performance of nucleic acid aptamers in applications such as analytical assays and clinical therapeutics. Divalent or multivalent nucleic acid aptamers are constructed by cross-linking monovalent aptamers together, such as through a polymer backbone, or by non-covalent cross-linking. Furthermore, some divalent or multivalent nucleic acid aptamers can also be prepared by tailoring and splicing based on recognition of their secondary structures or examination of their properties. In addition to weaving two or more nucleic acid aptamers, fragments of cleaved nucleic acid aptamers can be incorporated into activated probes by auxiliary sequences.

## 2. Paper-Based Analytical Methods

### 2.1. Carrier Materials of Paper-Based Analytical Methods

Paper-based analytical methods have been used in scientific research or application areas for many years, the first being applied in the 19th century with the invention of the pH measuring tool, litmus paper. The mass flow of the paper that was used in the test strip depends on many factors for paper structures, including specific surface area, capillary flow, pore size, paper thickness, and porosity [37]. Currently, the carrier materials of paper-based analyses include three categories: cellulose, modified or compounded cellulose, and non-cellulose.

#### 2.1.1. Cellulose

Cellulose is a hydrophilic polar polymer that can form strong non-covalent interactions with other charged or polar substances and has been used for paper-based direct enzyme-linked immunosorbent assays (ELISA) [38]. Cellulose paper-based assays are usually used to prepare chemical test strips, traditionally performed by saturating the paper carriers with the assay reagents and allowing them to dry. For example, a strip of paper with good water absorption impregnated with a pH indicator can be used as a portable pH assay strip after drying.

#### 2.1.2. Modified or Compounded Cellulose

Each glucose group in a cellulose macromolecular chain has three hydroxyl groups, which can be esterified with acids. During the esterification reaction with nitric acid, nitrocellulose is produced. A nitrocellulose with a cellulose backbone has amphoteric nitro groups, which generate strong non-covalent interactions with biomolecules. Nitrocellulose membranes are widely used in lateral flow immunoassay [39]. In addition, the compounding of cellulose with other materials allows the preparation of more versatile materials.

#### 2.1.3. Non-Cellulose

With the development of technology and interdisciplinary applications, the definition of “paper” has become very broad. Some materials are called paper-like materials due to their similarities with certain characteristics of paper. For example, polyimide paper-based functional materials can be prepared by a wet molding process in which polyimide fiber is used as a raw material. This non-cellulosic paper-based material is also known as a special polyimide paper with good processing adaptability, puncture resistance, and breathability, which is commonly used in transformers, motor coil winding, and turn-to-turn insulation. Furthermore, it is an essential class of temperature-resistant insulation materials in the manufacture of electrical equipment. In addition, glass filter paper, another non-cellulosic paper-based material, has good optical properties and biocompatibility, and there has been a breakthrough in glass microstructures with the continuous development of 3D printing technology.

### 2.2. Type of Paper-Based Analytical Methods

After more than a half-century of development, biosensors have been applied in various fields, including food detection, and have significantly impacted production and people’s lives [1]. Paper-based sensors have received much attention because of their multiple advantages, such as their light weight, flexibility, easy processing, cost-effectiveness, and low amount of environmental pollution [40,41]. The first paper-based sensor was paper chromatography invented by Martin and Singer, who were awarded the Nobel Prize in Chemistry in 1952. Another milestone in this field was the commercialization of pregnancy testing [42]. Paper-based biosensors can be divided into three main categories: dot filtration assay (DFA), lateral flow assay (LFA), and microfluidic paper-based analytical devices (μPADs). The paper-based sensors are summarized in Table 2.

#### 2.2.1. Test Strips

Test strips, also called paper-based sensors, are fast detection products characterized by low costs, short detection time, and simple operation. A paper-based sensor usually consists of three components: an identification element (biospecific identifier), a detection element (signal marker), and a signal conversion element (optical instrument or smartphone). These elements form a stand-alone test strip or a strip that has been enclosed in a plastic housing [43].

**Table 2:** Summary of paper-based sensors.

Paper-Based Sensors		Description	Characteristics	Advantages	Disadvantages	Ref.
Test strips	DFA	The DFA system is a plastic cartridge with a nitrocellulose membrane placed under the central hole of the cap, an absorbent paper placed at the bottom of the cartridge, and aptamers wrapped in the center of the membrane.	The test strips are divided into competitive- and sandwich-type test strips. The sandwich method is used for the detection of large analytes and analytes with multiple specific binding sites, and occasionally where there are two aptamers specific to a target. The competitive method is used for the detection of small analytes with a low molecular weight, analytes with single specific binding sites, and occasions in which there is only one aptamer specific to an analyte.	Concentrated color due to the dropwise addition of materials to be measured to the detection area. Circular detection area for other convenient subsequent processing and high detection sensitivity.	Not suitable for complex matrices because the test strips must be pretreated. Need for further confirmation in a positive test. Limited application; not yet applied on a large scale and for the market. Only for qualitative analysis. Quantitative analysis must be used in combination with other detection methods, and sensitivity is low.	[44]
	LFA	Targets and markers are adsorbed on aptamers. The microscopic changes in the detection line and quality control line are achieved by the specific recognition between aptamers and targets with the help of the characteristics of markers, such as color and fluorescence, thus achieving the detection of targets.		Low development cost. Easy to produce. Readily observed results.		[45]
Microfluidic Chips		The μPADs, referred to as paper chips, use paper as the carrier. The reaction occurs by controlling the direction and speed of liquid flow in the paper chip channels, thus realizing the purpose of building a “micro-laboratory” on a paper base.	The structure of paper-based microfluidic devices can be broadly classified into two-dimensional (2D) and three-dimensional (3D) devices. The main difference between them is in the structural design, functional integration, and multi-target and multi-step analysis capability.	Portability. Simple and reliable reaction system. High efficiency of nucleic acid molecule detection in complex systems with the combined use of 2D and 3D paper-based structures.	No complete laboratory supporting equipment or a related quality control system. Limited detection resolution due to the current microfabrication methods that cannot achieve high throughput and accurate quantification on a limited paper-based reaction space.	[46]

Among the test strips, Au NPs-based test strips generally come in two types: (1) those based on the longitudinal permeation of nitrocellulose membranes and used in the DFA and (2) those based on the lateral lamination of nitrocellulose membranes and used in the LFA.

##### Dot Filtration Assay

The DFA system is a plastic cartridge with a nitrocellulose membrane placed under the central hole of the cartridge lid and an absorbent paper at the bottom of the cartridge, with the aptamer encapsulated in the center of the membrane [47].

**Figure 1:**
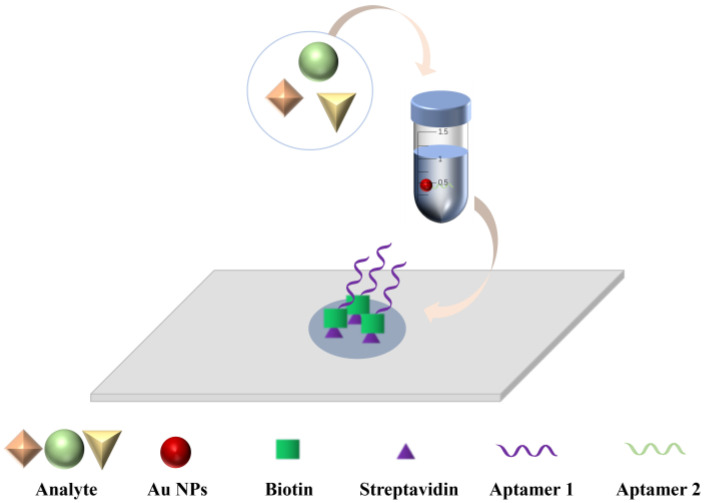
Schematic diagram of the Au-NPs based dot filtration assay.

The DFA methods with an aptamer as the recognition molecule are still in development. In Figure 1, we provide the principle of action of the aptamer-based dot filtration assay. In the double aptamer sandwich method, a sample is added dropwise to the aptamer area on the nitrocellulose membrane, and then an Au NPs reagent is added to label the test substance-aptamer complex. The test substance reacts specifically with the aptamer and binds to it, and the Au NPs-labeled test substance-aptamer complex is captured on the nitrocellulose membrane for color development. In this way, the substance to be tested is detected semi-quantitatively according to the shade of red.

##### Lateral Flow Assay

A typical LFA system consists of three elements: a recognition element, a response element, and a signal transduction element. All these elements are assembled on a paper strip system comprising five components: a sample pad, a binding pad, a membrane, an absorbent pad, and a backing plate [45,48]. The backing plate supports the strip assembly, and all other components overlap sequentially at their adjacent ends (2–3 mm overlap between every two components) to ensure liquid solution migration through the LFA strip [48,49]. The sample pad delivers the sample to the other components of the lateral flow strip system via a suction core. The binding buffer contains labeled biometric molecules that are released and bind to the passing liquid sample upon contact [50]. Test and control lines are set on the nitrocellulose membrane, which are critical for determining the sensitivity of the LFA, and an optimal and stable binding with the capture probe should be provided in the test line. Finally, the absorbent pad provides a capillary-based driving force that maintains the rate of the liquid sample migration through the membrane and prevents reflux [51].

The prototype design for LFAs was first reported by Plotz and Singer in 1956 [6]. It later attracted an explosion of interest when urine-based pregnancy tests became commercially available in the 1980s [52]. Due to the low developmental cost and simple production, LFAs have been widely used for on-demand applications. They have been applied in various fields, such as biomedicine, quality control, food safety, and environmental monitoring [53]. In addition, they can be used for a range of biological samples, including urine [54], saliva [55], sweat [56], serum [57], plasma, and blood [58]. There are two primary types of LFAs: sandwich and competition methods [50]. The sandwich method is preferred for detecting large analytes with multiple specific binding sites, and in occasions when there are two aptamers specific to a target. The competition method is used for the detection of small analytes with low molecular weights and only a single specific binding site, and occasions when there is only one aptamer specific to a target [59].

##### Sandwich-Type LFA Test Strips

The sandwich LFA is a typical LFA using a pair of aptamers, with the primary aptamer immobilized on the test line as a capture probe and a labeled biotinylated secondary aptamer dispersed on the conjugate pad as a detection probe [1,30]. Streptavidin was immobilized on the control line as a control probe in some tests [60,61]. When the analyte was added to the sample pad, it was initially bound to the labeled aptamer [1]. The formed complex was then captured by the primary aptamer, resulting in a sandwich-structure complex on the test line. Through the specific binding reaction between streptavidin and biotin, streptavidin captured the excess labeled biotinylated secondary aptamer for producing retention signals. As a result, two feature bands, both the test line and control line, indicate a positive result, while a single feature band only on the control line indicates a negative result [61].

**Figure 2:**
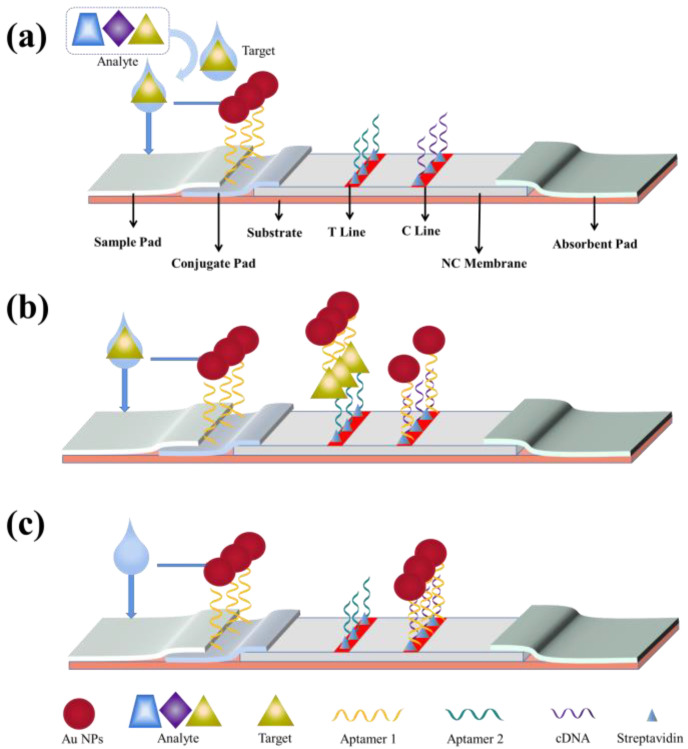
Schematic diagram for the operation principle of a typical sandwich-type test strip. (**a**) Sandwich-type lateral flow test strip diagram. (**b**) Reactions occurring in the sandwich-type lateral flow test strip in the presence of the target. (**c**) Reactions occurring in the sandwich-type lateral flow assay in the presence of no target. C line: control line; T line: test line.

As shown in Figure 2, the first aptamer, aptamer 1, is connected to Au NPs and the formed complex is placed on the binding pad. The second aptamer, aptamer 2, is immobilized on the test line by streptavidin, and the complementary cDNA of aptamer 1 is immobilized on the test line by streptavidin [62]. When there is a target in the sample, after the target-aptamer 1 binding on the binding pad, the target-aptamer 1 complex encounters aptamer 2 and binds to it on the test line, which forms a Au NPs-aptamer 1-target-aptamer 2 complex and develops a color on the test line. When there is no target in the sample, the target-aptamer binding does not take place, and the Au NPs-aptamer 1 complex is fixed on the control line by the complementary binding between aptamer 1 and cDNA, which results in a red color.

##### Competitive-Type LFA Test Strips

When target-aptamer binding occurs, the aptamer undergoes conformational changes to form specific structures, such as hairpins, stem loops, or G-quadruplexes, resulting in better target-aptamer binding [63]. When certain regions of an aptamer sequence undergo structural changes, Watson-Crick base pairing between an aptamer and a single complementary sequence bound to the aptamer may be disrupted, which leads to target-induced dissociation [64]. Therefore, in the presence of both a complementary sequence and a target in a sample, the target competes with the complementary oligonucleotide sequence for recognition by the aptamer (immobilized on the test line), providing a weak or no signal. In contrast, in the absence of the target analyte, the aptamer-reporter complex can be readily captured by the complementary sequence, which leads to a strong band on the test line [30].

**Figure 3:**
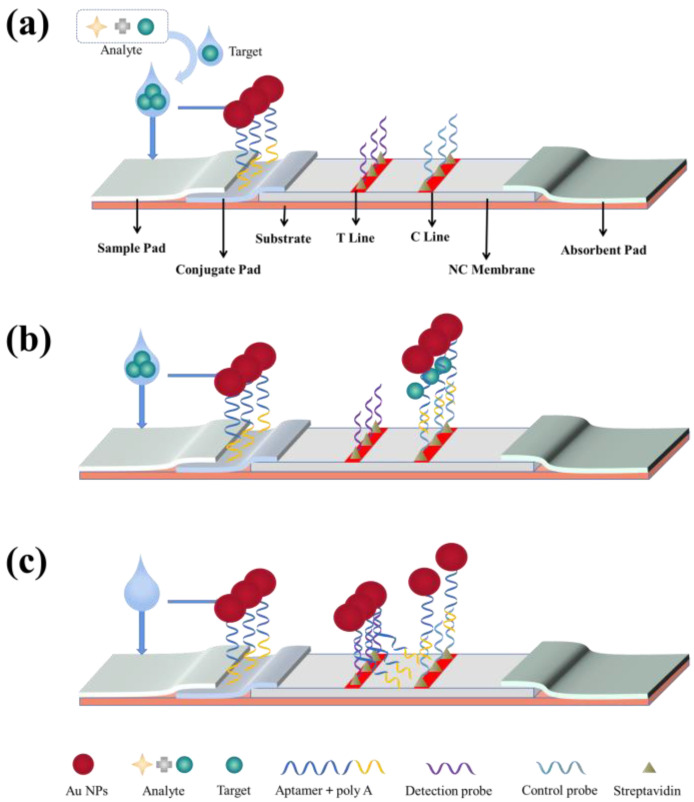
Schematic diagram of the operation principle of a typical competitive-type test strip. (**a**) Competitive lateral flow test strip. (**b**) Reactions occurring in a competitive lateral flow test strip in the presence of the target. (**c**) Reactions occurring in the competitive lateral flow test strip in the presence of no target.

As shown in Figure 3, the aptamer, poly (A), and Au NPs are linked to form an Au NPs-aptamer-poly (A) complex that is added onto the binding pad. After binding of the target to the aptamer, the structure of the aptamer is changed. Hence, the formed complex is fixed on the control line by the binding of poly (A) to poly (T) instead of binding to the cDNA on the test line, thus developing a color on the control line. When there is no target in the sample, the target-aptamer binding does not occur, and the structure of the aptamer remains unchanged, thus securing the cDNA on the test line and developing a color there [30].

For example, to detect trace amounts of aflatoxin B1 (AFB1) in food, Chao et al. synthesized an anthocyanin 5 (Cy5, a fluorophore)-conjugated DNA aptamer for designing a competitive (aptamer) Apt-LFA. They immobilized the complementary sequence on the test line for competitive binding to the aptamer [31]. Semi-quantitative detection was achieved by comparing the fluorescence intensity of the test and control lines.

#### 2.2.2. Microfluidic Paper-Based Analytical Devices

Microfluidic chip technology is a chip containing a tiny and precise channel structure as a carrier, which integrates the preparation, dilution, reaction, separation, detection, and analysis of trace samples onto a chip with an area of a few square centimeters [65]. The reaction and product analyses are based on the sample solution flow in the microchannels of a suitable chip [66].

In 2007, Whitesides’ team first proposed the concept of the microfluidic paper chip [32]. This paper chip can be combined with various optical and electrochemical analyses and immunoassays and has been rapidly developed in environmental monitoring, clinical medicine, and food safety.

**Figure 4:**
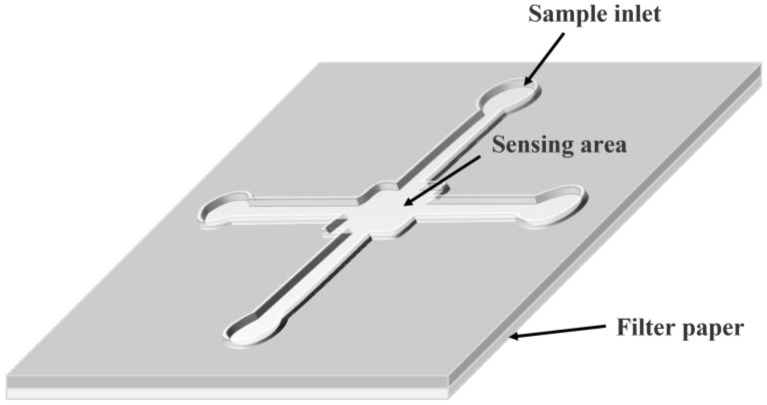
Schematic diagram of a paper-based microfluidic chip.

Microfluidic paper-based analytical devices (μPADs), hereinafter referred to as paper chips, are a new type of fluid handling and analysis system with great application and development potential that has become increasingly popular recently. As shown in Figure 4, the reaction is induced by controlling the flow direction and rate of the liquid in a paper chip channel, thereby realizing the purpose of constructing a “micro lab” on a paper base. This technology is integrated, miniaturized, and achieves high throughput, with the advantages of low cost, good flexibility, and biocompatibility of the paper base [67].

The 2D-μPADs are used to perform multiple simultaneous measurements in the same horizontal dimension. Although 2D-μPADs have the advantages of portability and the use of a simple and reliable reaction system, they are limited in their ability to cope with complex nucleic acid reaction systems in multiple dimensions and often can detect only single-target nucleic acid molecules. To guide the reaction system to flow horizontally in various directions and to achieve functions such as multi-target analysis and sequential reagent delivery in detecting nucleic acid molecules, more researchers are shifting their focus to microfluidic analysis with stereoscopic 3D structures. 3D-μPADs take greater advantage of the folding, bending, and twisting properties of the paper substrate to realize a transformation from two to three dimensions, and the increased dimensionality allows for the connection of microfluidic channels between different layers of the paper substrate, thus forming a multi-layer fluid network for the simultaneous and rapid quantification of different analytes in multiple detection areas. Compared with 2D-μPADs, 3D-μPADs provide more convenient operations, such as multiplexed and multi-step analyses. Furthermore, the nucleic acid molecule detection efficiency in complex systems can be improved by the combined use of 2D and 3D paper-based structures.

### 2.3. Detection Method of the Paper-Based Analytical Assay

The main sensing strategies currently used in combination with paper-based methods are colorimetric, fluorescence, electrochemical, and photothermal detection.

#### 2.3.1. Colorimetric Detection

Colorimetric analysis is one of the most common and simplest methods of signal output. It is also one of the most classical analytical methods used in chemistry and biology experiments. The colorimetric method is widely used in analytical testing because it does not require advanced equipment, is inexpensive, and can obtain intuitive results conveniently and quickly [68]. On a detection platform, colorimetric analysis is based on a colorimetric reaction that transforms the measured components in the sample into colored compounds. Qualitative research using colorimetric methods is carried out according to the change of color, and the quantitative or semi-quantitative analyses performed by colorimetric methods are based on the relationship between the color shade and the concentration of the measured components. The change in color can be observed directly by the naked eye, and pictures can be obtained using photography and image scanners and then analyzed through relevant software [66]. This analysis can be used for food safety testing, such as qualitative analysis of food components, the detection of pathogenic bacteria, heavy metal residues, and food additives.

However, colorimetric analysis also has certain shortcomings: (1) uneven color development, which is caused by uneven pore structure of the paper used; and (2) many interference factors, such as the base color of the paper, the color of the reagent, and interference from light.

#### 2.3.2. Fluorescence Detection

The principle of fluorescence detection is that certain fluorescent groups can produce fluorescence under specific lasers. The aptamer or target is bound to the fluorescent group, and its properties are observed under specific light. In recent years, fluorescence detection has been widely used in paper chip technology due to its high sensitivity [69]. However, this detection method is subject to the interference of background fluorescence when using fluorescence equipment. During the finishing process in the manufacturing process of paper, some fluorescent agents are added, which can sometimes have a minor impact on the detection results [70]. Therefore, when high precision is required, the paper should be treated in advance to minimize the impact of background fluorescence on detection results.

**Figure 5:**
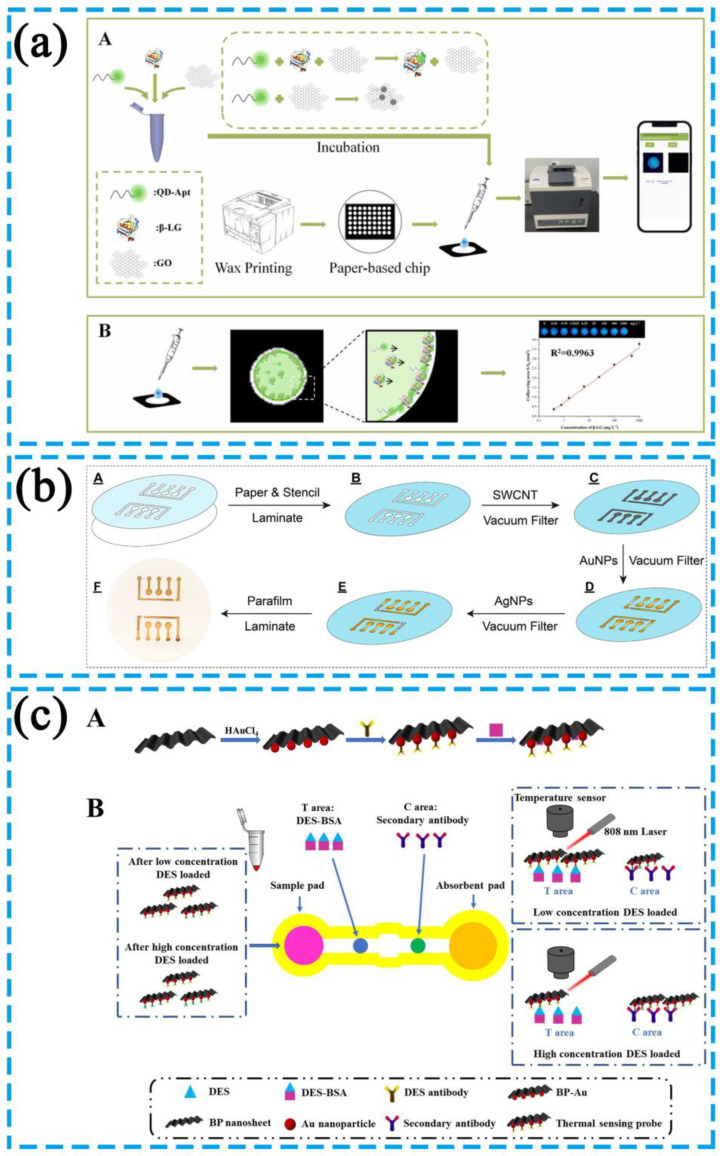
(**a**) Diagram of a fluorescence resonance energy transfer (FRET)-based biosensor. Reprinted with permission from Ref. [71]. Copyright 2023, Elsevier B.V. (**b**) Diagram of a rapid detection method of cancer cells by the photothermal effect of GO in permeabilized test strips (DFA). Reprinted with permission from Ref. [72]. Copyright 2021, American Chemical Society. (**c**) Diagram of a photothermal-sensing microfluidic paper-based analytical chip (PT-Chip). Reprinted with permission from Ref. [76]. Copyright 2023, Elsevier B.V.

As shown in Figure 5a, Yang et al. utilized the quantum dot-aptamer-graphene oxide (QD-Apt-GO) fluorescence system to produce a coffee ring effect on the filter paper [71]. Owing to the rapid evaporation rate at the liquid edge, the force derived from outward flow transported the suspended particles from the center to the outer edge, thus forming a coffee ring. The fluorescence signal changed according to the FRET principle of QD-Apt-GO. The beta-lactoglobulin (β-LG) in the system could bind specifically to the QD-Apt complex instead of attaching to GO to form a QD-Apt-β-LG complex, thus increasing the fluorescence intensity. When the target is added into the detection zone, the outward capillary flow in droplets carried the solution to the outer edge, thus resulting in a coffee ring; quantitative fluorescence detection was subsequently performed in this region.

#### 2.3.3. Electrochemical Detection

The principle of the electrochemical method is to use electrochemical reactions to produce signal changes that can be well-coupled with paper chips owing to their advantages of high sensitivity, good accuracy, and relatively simple instrument operations. Electrochemical detection effectively avoids interference caused by uneven color reactions in the detection area of paper devices. Since only the electrical signals generated by the chemical reaction in the detection area of a paper are collected, electrochemical detection has a broader range of applications compared with colorimetric analysis. It can be used for accurate testing of gas samples and biological molecules [70]. However, electrochemical detection also has non-negligible disadvantages, the signals decay and the electrodes need to be replaced periodically as the electrodes physically adsorb the liquid in their vicinity.

As shown in Figure 5b, Liu et al. constructed aptamer-modified multiplex paper electrochemical devices (mPEDs) that can detect multiple small molecules in a complex sample in a few seconds consisting of a silver pseudo-reference electrode (RE), a gold counter electrode (CE), three gold working electrodes (WE1-3), and a gold circuit network [72]. A single-walled carbon nanotube (SWCNT) solution was first filtered onto a paper substrate under vacuum to build the conductive layer. The Au NPs solution was then added to the paper substrate in a similar manner to form a gold thin film on the SWCNT. Silver nanoparticles (Ag NPs) were vacuum filtered onto the designated area to generate a silver pseudo-reference electrode. Finally, a para-film laminated polyethylene terephthalate (PET) bottom mask was laminated to the bottom of the paper substrate to complete the device.

#### 2.3.4. Photothermal Detection

For some nanomaterials, light energy can be transformed into heat energy after radiation by photons of different wavelengths, which is reflected in the rise of the nanomaterial temperature. In the photothermal spectrum, since near infrared (NIR) light has the longest wavelength and acts on free electrons for a longer time than lights in other wavelength ranges, electrons radiated by it can vibrate more rapidly and generate more thermal energy. Therefore, NIR light can induce the strongest photothermal effect. The relatively common nanomaterials used for photothermal-sensing include precious metal nanomaterials (such as Au NPs [73] and Ag NPs), carbon nanomaterials (such as graphene nanomaterials), transition metal chalcogenide nanomaterials (such as copper sulfide [74] and molybdenum disulfide nanomaterials), and organic dye nanomaterials (such as indocyanine green and Prussian blue nanomaterials [75]). After laser irradiation, the nanomaterials release heat energy, generating a readable signal of the temperature change.

In recent years, detection methods based on the photothermal effect of Au NPs have emerged one after another. Qin et al. first used the Au NPs-based photothermal method on immunochromatography test strips. Compared with the traditional colorimetric method, the limit of detection was increased by 32-fold [77].

However, it is mostly applied with antibody test strips, and no aptamer-based test strips with the photothermal application have appeared. Several examples of the photothermal effect applied to antibody-based test strips follow. As shown in Figure 5c, Wang et al. developed a photothermal-sensing microfluidic paper-based analytical chip (PT-Chip) to detect diethylstilbestrol (DES) [76]. A composite of black phosphorus nanosheets and gold nanoparticles (BP-Au), which has excellent photothermal properties, was used as a signal indicator. Based on the principle of competitive immunostaining, an antigen that can competitively bind to the photothermal probe against DES was placed on the detection area to capture the probe. The detection area was then irradiated by a laser with an 808-nm wavelength, and the amount of DES was obtained by analyzing the temperature change profile of the test area. This approach also provides a reference for the development of paper-based photothermal detection device based on aptamers.

## 3. Applications of the Aptamer and Paper-Based Analytical Methods

Taking the widely used LFAs as examples, this section briefly describes the application of LFA for various analytes using aptamers as recognition elements. We divide the applications of aptamers into the detections of cell-based, protein-based, small molecule-based, and ionic substances.

### 3.1. Cell-like Substance Detection

It should be noted that there is no uniform definition of a cell; the more common statement is that a cell is an organism’s basic structural and functional unit. All organisms, except viruses, are known to be composed of cells. Cells have the basic structure of a cell membrane, cytoplasm, and nucleus. Cell-like substances refer to substances with a cellular network.

For the application of LFA on cell-like substance detection, Wu et al. screened two different aptamers specific to the outer membrane of *E. coli* O157:H7. One of the aptamers was used for magnetic bead enrichment, and the other was used as a signal reporter for this pathogen [44]. The latter aptamer was an ssDNA amplified by isothermal strand displacement amplification (SDA) and further constructed for an LFA determination of *E. coli* O157:H7. The unidirectional SDA was first performed using the enriched aptamer on magnetic beads anchored to the outer membrane of *E. coli* O157:H7 as a template, and the probe-Au NPs complex was placed on the binding pad. The amplified ssDNA was captured by a complementary reaction between the probe and ssDNA to form an ssDNA-probe-Au NPs complex. The formed complex was then captured on the test line by the complementary binding between the ternary complex and a DNA immobilized on the test line, and that resulted in a red band on the test line. As the probe part of the excess probe-Au NPs complexes complementarily bound to a DNA immobilized on the control line, a second red band was developed there. In the absence of the *E. coli* O157:H7, the amplification process failed, there was no free ssDNA, and therefore no color development on the test line.

**Figure 6:**
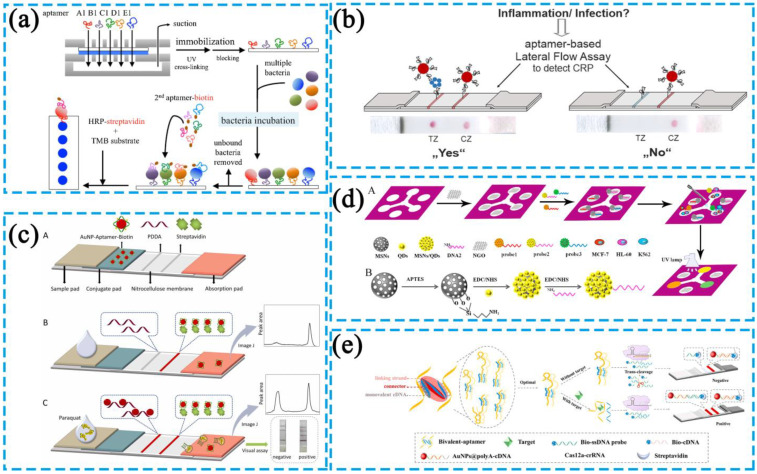
(**a**) Diagram of a rapid detection method for multiple bacteria in paper-based microfluidic chips. Reprinted with permission from Ref. [78]. Copyright 2019, Elsevier B.V. (**b**) Diagram of a rapid detection method of C-reactive protein levels in human patients in a lateral flow assay. Reprinted with permission from Ref. [80]. Copyright 2020, American Chemical Society. (**c**) Diagram of a rapid detection method for paraquat in a lateral flow assay. Reprinted with permission from Ref. [81]. Copyright 2022, Elsevier B.V. (**d**) Diagram of a rapid detection method for cancer cells in paper-based microfluidic chips. Reprinted with permission from Ref. [79]. Copyright 2016, Elsevier B.V. (**e**) Diagram of a rapid detection method for CRISPR/Cas12a small molecules in a lateral flow assay. Reprinted with permission from Ref. [82]. Copyright 2022, Elsevier B.V.

As shown in Figure 6a, in the microfluidic system, Wang et al. injected the selected aptamer 1 into the loading chamber, which was then transferred to and immobilized on the NC membrane by a suction pump [78]. Bacteria were then added into the NC membrane and allowed to bind aptamer 1. The biotin-bound aptamer 2 was subsequently added, which attached to the target bacteria to form a complex. Next, the streptavidin-horseradish peroxidase (HRP) was added and connected to the complex. The 3,3′, 5,5′-tetramethyl benzidine (TMB) was finally added to react with the HRP portions bound on the NC membrane. The change in blue color indicated the presence of the target bacteria.

As shown in Figure 6d, Liang et al. added GO and a fluorescent probe consisting of an aptamer to the corresponding area on a paper chip μ-PAD [79]. Various concentrations of cancer cells were then added for quantitative analysis using a UV lamp or a spectrofluorometer.

### 3.2. Protein-like Substance Detection

Proteins are spatially structured substances formed by the coiling and folding of a polypeptide chain composed of amino acids through dehydration condensation, which can be divided into animal proteins and vegetable proteins [83]. Protein is an essential component of all cells and tissues in the human body, and all parts of the human body require protein participation to function.

LFA has been widely used in protein-like substance detection. For example, as shown in Figure 6b, in a screening process, Phung et al. immobilized an aptamer with affinity to C-reactive protein (CRP) on a microarray chip [80]. The assay system was constructed in various ways, such as forward and sandwich formats, for optimizing different conditions. Based on the optimized conditions, an aptamer-based LFA was established to detect CRP levels in human patients. The target CRP was successively bound to two aptamer molecules located on the test and control zones of the LFA system in a sandwich-like manner. The sample pad of the LFA system contained a red Au NPs-aptamer conjugate (Au NPs-Apt1), and the amino-modified aptamer, Apt1, and the amino-modified complementary short oligonucleotide (cDNA) were immobilized on the test zone and control zone of the nitrocellulose membrane, respectively. When the analyte CRP was present in the sample at a certain concentration, CRP bound to the conjugate on the sample pad and formed a CRP-Apt1-Au NPs complex, which was then captured by the Apt1 on the test zone. Finally, an Apt1-CRP-Apt1-Au NPs sandwich-like structure was formed on the test zone and a red color developed. The unbound Au NPs-Apt1 complexes further migrated to the control zone. Even without the target CRP, Au NPs-Apt1s hybridized with the complementary short oligonucleotide immobilized in the control zone, forming a second red zone on the membrane, thus indicating the effectiveness of LFA detection.

### 3.3. Detection of Low Molecular Weight Substances

Low molecular weight substances are natural compounds with low molecular weights. These are usually biofunctional molecules with molecular weights of less than 1000 Dalton and small molecules that are simple monomeric substances, including nucleotides, amino acids, glucose, and fatty acids.

In an example of using an LFA to detect low molecular weight substances, Liu et al. added the cationic polyelectrolyte poly (diallyl dimethylammonium chloride) (PDDA) onto a test line, which captured Au NPs by strong electrostatic interactions, thus establishing a simple aptamer-based chromatographic strip for rapid visual detection of paraquat, as shown in Figure 6c [81]. During the assay, the sample was first added to the sample pad, and the target in the sample (paraquat) reacted with the Au NPs-aptamer complex in the binding buffer to provide free Au NPs, which were captured by PDDA and developed a red color on the test line of the nitrocellulose membrane. In the absence of the target, the portion of the Au NPs-aptamer complex could not be captured by PDDA, and the test line remained unchanged. The immobilized streptavidin on the control line was bound specifically to the excess Au NPs-aptamer complex, and a red color appeared on the control line.

Wu et al. constructed a zearalenone (ZEN) aptamer-based LFA that worked by competitive binding between a DNA on the test line (DNA 1) and ZEN in the sample to the Au NPs-Apt [14]. In the absence of the target (ZEN), Au NPs-Apt is complementarily bound to DNA 1, resulting in a red color on the test line. However, in the presence of ZEN, the aptamer portion of the Au NPs-Apt bound to ZEN instead of binding to DNA 1, and therefore, no color developed on the test line. Au NPs-Apt is also bound to another DNA sequence (DNA 2) on the quality control line (control line). Regardless of the presence of ZEN in the sample solution, the Au NPs-Apt is always bound to DNA 2, generating a red color on the control line.

As shown in Figure 6e, Li et al. constructed a bivalent aptamer consisting of two aptamer strands and a linker strand, where the linker strand consisted of two short DNA fragments (cDNAs) that partially complemented the aptamer and a T-base repeat segment connecting the two cDNAs [82]. In the absence of targets, a molecular hybridization occurred between the bivalent aptamer and Cas12a-crRNA, activating the trans-cleavage activity of Cas12a to cleave biotin-modified ssDNAs (Bio-ssDNAs), and the cleaved Bio-ssDNA strands could not bind to the Au NPs@polyA-DNA probes. The probe complexes could not be captured by SA on the T-line or develop a color there. In contrast, upon ATP binding, the molecular hybridization did not occur, and the trans-cleavage activity of Cas12a was therefore not activated. Bio-ssDNAs bound to the Au NPs@polyA-DNA probes and developed a color on the T-line.

### 3.4. Ion-like Substance Detection

Ions, like molecules and atoms, are also fundamental particles that compose matter. Ions are divided into cations and anions. Substances made up of ions, such as sodium chloride, barium sulfate, and sodium hydroxide, can ionize anions and cations in a solution or in molten state.

As a practical application example of LFA in ion-like substance detection, Wu et al. constructed a competitive LFA by applying an aptamer-based fluorescence quenching mechanism [18]. When the target mercury ion (Hg^2+^) was present, it bound to the aptamer portion of the Au NPs-Apt and occupied the binding site, so the Au NPs-Apt-target complex did not bind to the probe coated on fluorescent microspheres on the test line. The fluorescence on the test line therefore could not be quenched completely and produced a signal. When there was no target in the sample, the Au NPs-Apt complex hybridized with the probe on the test line and quenched the fluorescence. Throughout this process, the Au NPs-Apt complex bound to bovine serum albumin-fluorescent microspheres on the control line regardless of whether there was a target in the test solution, so the fluorescence on the control line was quenched by the aptamer in both situations.

Qian et al. prepared an electrochemical paper-based chip using the strong affinity of aptamers for cadmium and lead ions [84]. The Au NPs were added to the prepared electrochemical paper chip, and the cDNAs were immobilized on the chip by Au NPs. Then, the aptamer solution was added to form a complementary aptamer-cDNA double-stranded structure. In the absence of targets, the amides of cadmium and lead ions were modified by ferrocene and methylene blue to produce a high-intensity current signal. The addition of target cadmium and lead ions disrupted the aptamer-cDNA double-stranded structure, and the cleaning of the paper chip could weaken the electrochemical signal. The concentrations of target cadmium and lead ions could be quantified by measuring the changes in the electrochemical signals of methylene blue and ferrocene modified on aptamers.

A microfluidic aptamer sensor capable of detecting Hg and Pb ions simultaneously was developed by Huang et al. [85]. The GO was used as the quencher, and an octanoic acid solution was used as the reagent. After labeling the aptamer sequence with FAM and HEX fluorescent dyes and mixing it well with the GO solution, it was injected into another port. Meanwhile, the solution containing Hg or Pb ions was injected into the other port. During the subsequent mixing process, aptamers bind to Hg or Pb ions and undergo fluorescence resonance energy transfer (FRET); the presence of Hg or Pb ions can be detected by measuring the change in the fluorescence intensity of the GO/aptamer suspension.

## 4. Future Prospects

**Figure 7:**
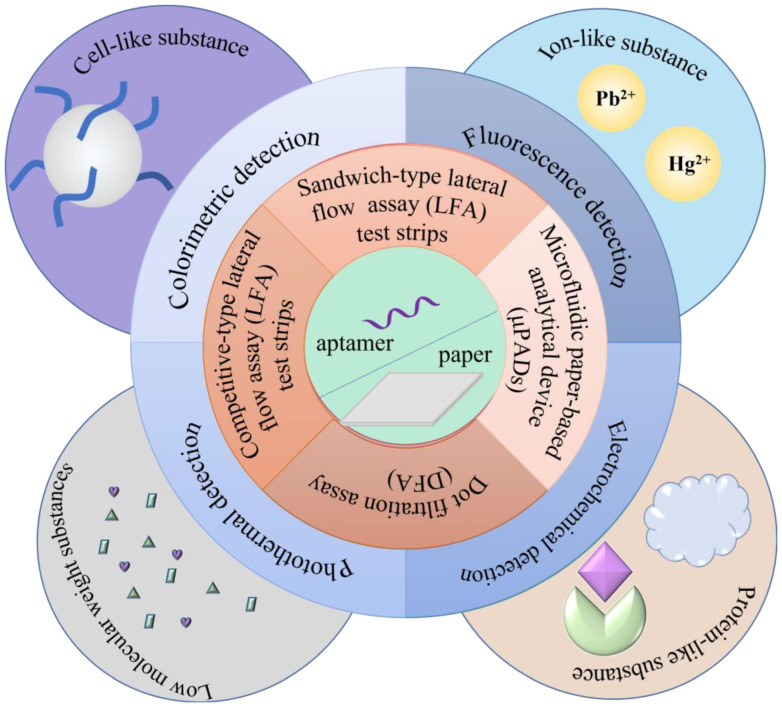
Overview of the use of aptamer in paper-based sensors.

Paper-based sensors are suitable for mass production as they can be paired with various existing instruments. The cost of synthesizing these successfully screened aptamers is significantly lower than that of antibodies [86], which can be utilized as a very cheap and efficient technique. Moreover, due to the inherent nature of nucleic acids, aptamers can also recover their conformation after denaturation, allowing them to be better preserved [87]. Soon, paper-based biosensors will be followed by enhanced sensitivity and multi-molecule detection. The use of aptamers in paper-based sensors are summarized in Figure 7.

Despite the outstanding advantages of aptamers, the practical applications of aptamer-based paper sensors have yet to be commercialized. Only a few aptamers have been successfully used in paper-based sensors. One reason for this is there are still few aptamers that can be employed in the development of relevant sensors. Second, the matrix in which the sample is present would influence the response of aptamer-based paper sensors. So, most of the pre-treatment work for their methods still requires specialized laboratory-type operations. Third, fewer successful paper-based sensors are prepared using aptamers, which still exist mainly in the laboratory. The almost aptamer-based sensing methods reported so far are somewhat complex or complicated to run as sensors, which usually raises the coefficient of variation and is not user-friendly in the field [88].

In the future, many problems must be solved along with the further development of aptamer applications. Moreover, solving these problems will become an essential part of future research. One is the possibility of modifying the aptamers, which can be achieved to improve the sensitivity and specificity [1]. Then, researchers must target the analytes with detection needs in the marketplace to screen reliable aptamers for the development of suitable detection devices [88]. Third, using each line of aptamers in the system for analysis, multiple assays should be developed to enable low-cost and rapid quantitative analysis [1]. Fourth, the application of small instruments to paper-based sensors can improve the quantification of analytes, achieving higher sensitivity and lower detection limits.

## 5. Conclusions

This paper reviewed the development of aptamers and their applications on paper-based sensors. Various SELEX screening methods were utilized for aptamer screening. Aptamers were identified for different target analytes, including small organic compounds, proteins, and biosensors [89]. After an initial screening process, aptamers generally needed to be further optimized through truncation, mutations, and chemical modifications.

Paper-based sensors using aptamers as recognition elements have only been around for a decade or so, but they have been developed rapidly for uses in rapid detection. They can be used to detect various target analytes, such as cells, protein substances, and small molecules. In addition, with the development of paper-based sensors, the functionality of the paper-based substrate and other components of the sensors have been dramatically improved [90]. However, sensors based on aptamers and papers are still only in the laboratory and have not yet entered into production for practical applications.

Although there is great room for improvement, we believe that aptamer and paper-based sensors will eventually become a mature method for rapid detection and will respond to the challenges that existing technologies cannot address.

## Data Availability

Not applicable.
